# Transcriptomic characterization of the human segmental endotoxin challenge model

**DOI:** 10.1038/s41598-024-51547-0

**Published:** 2024-01-19

**Authors:** Christina Gress, Tobias Litzenburger, Ramona Schmid, Ke Xiao, Florian Heissig, Meike Muller, Abhya Gupta, Jens M. Hohlfeld

**Affiliations:** 1 Fraunhofer Institute for Toxicology and Experimental Medicine ITEM, Clinical Airway Research, 30625 Hannover, Germany; 2 German Center for Lung Research (DZL-BREATH), Hannover, Germany; 3 Boehringer Ingelheim Pharma GmbH & Co. KG, Biberach an der Riss, Germany; 4 Boehringer Ingelheim International GmbH, Biberach an der Riss, Germany; 5 Hannover Medical School, Department of Respiratory Medicine and Infectious Disease, Hannover, Germany

**Keywords:** Experimental models of disease, RNA sequencing, Biomarkers, Gene regulation in immune cells, Inflammation

## Abstract

Segmental instillation of lipopolysaccharide (LPS) by bronchoscopy safely induces transient airway inflammation in human lungs. This model enables investigation of pulmonary inflammatory mechanisms as well as pharmacodynamic analysis of investigational drugs. The aim of this work was to describe the transcriptomic profile of human segmental LPS challenge with contextualization to major respiratory diseases. Pre-challenge bronchoalveolar lavage (BAL) fluid and biopsies were sampled from 28 smoking, healthy participants, followed by segmental instillation of LPS and saline as control. Twenty-four hours post instillation, BAL and biopsies were collected from challenged lung segments. Total RNA of cells from BAL and biopsy samples were sequenced and analysed for differentially expressed genes (DEGs). After challenge with LPS compared with saline, 6316 DEGs were upregulated and 241 were downregulated in BAL, but only one DEG was downregulated in biopsy samples. Upregulated DEGs in BAL were related to molecular functions such as “*Inflammatory response”* or “*chemokine receptor activity”*, and upregulated pro-inflammatory pathways such as “*Wnt-"/“Ras-"/“JAK-STAT” “-signaling pathway”*. Furthermore, the segmental LPS challenge model resembled aspects of the five most prevalent respiratory diseases chronic obstructive pulmonary disease (COPD), asthma, pneumonia, tuberculosis and lung cancer and featured similarities with acute exacerbations in COPD (AECOPD) and community-acquired pneumonia. Overall, our study provides extensive information about the transcriptomic profile from BAL cells and mucosal biopsies following LPS challenge in healthy smokers. It expands the knowledge about the LPS challenge model providing potential overlap with respiratory diseases in general and infection-triggered respiratory insults such as AECOPD in particular.

## Introduction

Major respiratory diseases such as community-acquired pneumonia (CAP) and acute exacerbations of chronic obstructive pulmonary disease (COPD; AECOPD) are among the most common causes of acute hospitalization^1^. CAP is caused by bacterial infections and is characterized by abrupt onset of illness accompanied by clinical symptoms such as fever, chills, malaise, cough, and dyspnoea^2–4^. AECOPD are episodes of worsening COPD symptoms such as dyspnoea, cough, and sputum production driven by airway inflammation^5,6^, often caused by bacterial or viral infections^7^. Accordingly, pathogen-derived lipopolysaccharides (LPS) may be central drivers in bacterial pneumonia and COPD exacerbations. LPS is a ubiquitous, potent, and well-known endotoxin of the outer membrane of gram-negative bacteria that signals via toll-like receptor 4 and activates a variety of intracellular signalling pathways leading to a profound cellular infiltration^8^.

Translation of preclinical findings to humans for providing target engagement, proof-of-principle or -mechanism (pharmacological proof of principle), and proof-of-clinical concept is valuable for early clinical stages of drug development. There are major differences between animal and human lungs regarding anatomy, physiology, as well as cell and molecular biology that limit translation of preclinical findings, making an early human proof-of-concept valuable^9^. While there is some justification to start first-in-human trials in patients, healthy volunteer studies remain the current practice. However, non-diseased organs in healthy volunteers do not exhibit specific pathway activation, or relevant cell migration and inflammation. Human challenge models can be used to induce distinct changes and mimic specific reactions in respective organs resembling features of disease^10,11^.

Segmental challenge with LPS to the lung is a well-established method to induce transient airway inflammation in healthy volunteers^10^. When instilled in lung segment, it uses a well-controlled design with saline instillation in a second lung segment as an internal control^12^. In contrast to inhalative LPS challenge, which stimulates the whole lung, only a fraction of the LPS dose is required for segmental application and lung sampling can be taken at the time of instillation. Bronchoscopy with bronchoalveolar lavage (BAL), bronchial mucosal brushing and mucosal biopsies allow sampling of human airway and inflammatory cells from different airway locations for RNA analysis amongst others. LPS challenge in humans mimics some aspects of COPD and exacerbations thereof, in particular neutrophil influx^13,14^. However, insights into immune regulation and pathway activation occurring in (AE)COPD or CAP in relation to the LPS challenge model are limited and did not include comprehensive analysis of transcriptomic data.

Transcriptomic data can be obtained with small amounts of sample material (≥ 50 pg RNA). Depending on the protocol, it allows generation of gene expression profiles of protein-coding as well as non-protein-coding genes in a given sample. In recent years, RNA-sequencing (RNA-seq) technology has developed rapidly, enabling the data-driven analysis of differential gene expression.

The aim of this work was to generate and describe the transcriptomic profile of the human segmental LPS challenge model using cells derived from BAL and mucosal biopsies. Furthermore, transcriptomic data were compared and contextualized with major respiratory diseases.

## Methods

### Study design

Transcriptomic characterization was performed with samples from the placebo group of a monocentre, randomized, double-blind, placebo-controlled, parallel-group, phase I trial in healthy subjects to assess pharmacodynamic effects and safety of 4 weeks' oral administration of bradykinin 1 receptor (B1R) antagonist (BI 1026706) on segmental endotoxin-induced inflammatory response (NCT02657408). Efficacy and safety data have been previously reported elsewhere^15^.

### Subjects

Healthy males and females not of childbearing potential aged 18–65 years with a body mass index (BMI) of 18.5–29.9 kg/m^2^, and normal lung function (forced expiratory volume in 1 s (FEV_1_) of > 80% of predicted normal and FEV_1_/forced vital capacity (FVC) ratio of > 70%) at the screening visit were eligible for inclusion. Participants were current smokers with a smoking history of at least 1 pack-year and at least 1 cigarette per day in the previous year, confirmed by positive cotinine test. Subjects were excluded if they had a history of any other clinically relevant disease, suffered from a lower respiratory tract infection in the previous 4 weeks, or had contraindications to medications used for bronchoscopy. Written informed consent was obtained from all subjects after they were fully informed about all trial-related aspects before any study-related procedures.

### Sample collection

**Figure 1:**
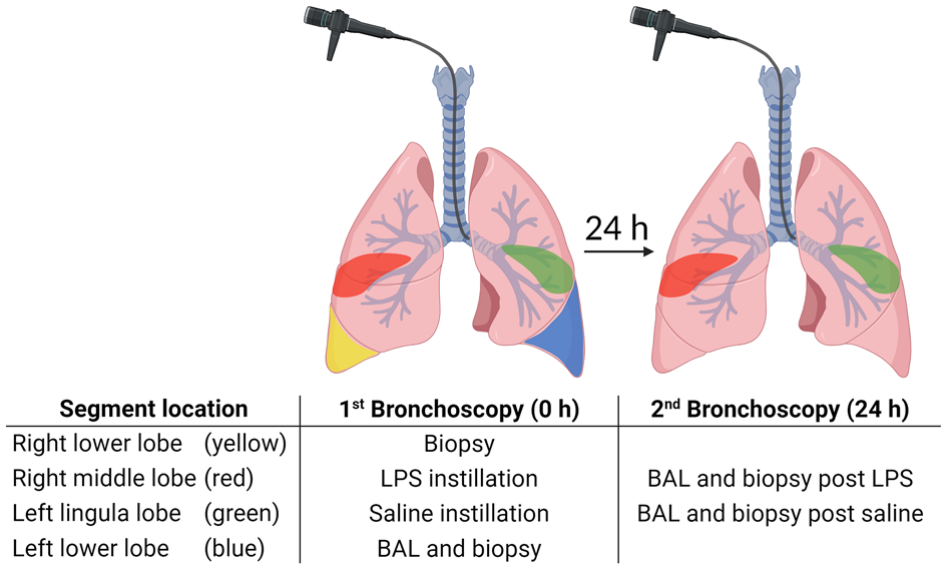
Study design and sampling scheme. *BAL* bronchoalveolar lavage *LPS* lipopolysaccharide. Created with BioRender.com.

Subjects underwent a first bronchoscopy (28 ± 2 days after placebo treatment) to collect pre-challenge baseline BAL from a segment of the left lower lobe using 100 mL of pre-warmed saline. Two baseline mucosal biopsies from the anterior segment of the left lower lobe and the right lower lobe were sampled. Segmental challenge with LPS (40 endotoxin units per kg body weight diluted in 10 mL of saline; endotoxin from E. coli Type O113; List Biological Laboratories Inc., Campbell, California, USA) was performed in the medial segment of the middle lobe and 10 mL saline (0.9%) was applied in the medial segment of the lingula as control. A second bronchoscopy was performed 24 hours later for collection of BAL and mucosal biopsies from the saline- and LPS-challenged lung segments^12^ (Fig. 1). Bronchoscopies were performed according to the guidelines for investigative bronchoscopies^16,17^. Details of the bronchoscopic procedure with BAL, biopsies, and segmental bronchial instillation have been described previously^18,19^.

### Processing of mucosal biopsies and BAL samples

Mucosal biopsies were assessed macroscopically during the bronchoscopy procedure to ensure the sampling was not only of mucoid material, immediately transferred into RNAlater RNA Stabilization Reagent (Qiagen, Venlo, The Netherlands), frozen at − 20 °C within 120 min overnight, and then transferred to − 80 °C for storage. Collected BAL samples were filtered (100 μm, BD Biosciences, Heidelberg, Germany), centrifuged (300 g, 10 min, 4 °C), and the cell pellet was resuspended in Dulbecco's phosphate-buffered saline. Total cell counts in BAL samples were determined by light microscopy after staining with trypan blue using a Neubauer chamber and calculated as absolute cell numbers normalized to BAL volume recovery [10^6^/mL]. Differential cell counts were determined by counting 800 cells microscopically on cytospins stained with Diff quick (RAL Diagnostics, Martillac, France). Because this technique cannot precisely differentiate between macrophages and monocytes, both were counted together as macrophages/monocytes. In addition, monocytes were determined separately by flow cytometry using granularity and expression of CD14 for identification as described previously^19^. Monocytes were then subtracted from macrophages/monocytes to derive the macrophage fraction. BAL cell samples were stabilized in RNAprotect Cell Reagent (Qiagen, Venlo, The Netherlands) and stored at − 80 °C. 

### Sequencing of human biopsy and BAL samples

Total RNA was isolated from all biopsy samples using the RNeasy Fibrous Tissue Mini Kit, and BAL samples using the RNeasy mini-Kit (Qiagen, Venlo, The Netherlands) according to the manufacturer’s instructions. RNA was eluted in RNase-free water and the concentration as well as the purity was determined via absorbance measurement using a NanoDrop device. Eluted RNA was stored at − 80 °C. Library preparation was performed using the TruSeq Stranded Total RNA Kit with Ribo-Zero Gold (Illumina, San Diego, USA) following the manufacturer’s instructions, and all samples were sequenced single-end and strand-specific on the HiSeq3000 (Illumina, San Diego, USA).

### Processing of RNA-seq raw data

Sequenced data were analysed using the Galaxy web platform (usegalaxy.eu)^20^. Default settings were used for the tool application, unless otherwise mentioned. For quality control *FastQC Galaxy Version 0.72* was applied to the raw data and reports were checked for “per base sequence quality”, “overrepresented sequences” and “adapter content”^21^. Data with poor quality in “per base sequence quality” or “adapter content” were excluded from further analysis. Data with “overrepresented sequences” were trimmed via *fastp Galaxy Version 0.20.1*^22^ and checked again for quality using *FastQC*. Reads were mapped to the human GRCh38 reference genome (https://www.gencodegenes.org/human/releases.html) using the Gencode main annotation file (gencode.v37) via *RNA Star Galaxy Version 2.7.8a*^23^. From the output with the mapped sequences, the number of reads per annotated genes was determined using *FeatureCounts Galaxy Version 2.0.1*^24^. To remove unwanted variation the control gene method *RUVSeq Galaxy Version 1.26.0* was applied to counted gene files^25^. Using *DESeq2 Galaxy Version 2.11.40.6* counts were normalized, principal component analysis (PCA) plots were created, and differential expression was calculated using unpaired sample analysis^21^. Differentially expressed genes (DEGs) were defined by the following criteria: adjusted p-value ≤ 0.05, BaseMean ≥ 2, and absolute log twofold change (|log2FC|) ≥ 1.

### Visualization of processed RNA-seq data

Gene names were determined in the manuscript based on ENSEMBL release 107 (July 2022)^26^. Data were visualized using *Volcano Plot Galaxy Version 0.0.5* and *heatmap2 Galaxy Version 3.0.1*. Gene set enrichment analysis was performed with highly differentially expressed genes (|log2FC|≥ 3, adjusted p-value ≤ 0.05) using *DAVID Analysis Wizard* *Version 2021* to determine enriched pathways (KEGG_pathways), biological processes (GOTERM_BP_DIRECT), and molecular functions (GOTERM_MF_DIRECT)^27,28^. Furthermore, highly differentially expressed genes (|log2FC|≥ 3, adjusted p-value ≤ 0.05) were annotated to the five most prevalent respiratory diseases (COPD, asthma, pneumonia, tuberculosis and lung cancer^29^) according to Ingenuity Knowledge Base^30^.

### Ethics approval and consent to participate

The protocol was approved by the Independent Ethics Committee of the trial site, Ethics Committee of Hannover Medical School, Hannover, Germany, and the German Federal Institute for Drugs and Medical Devices (BfArM). The study was conducted at the Fraunhofer Institute for Toxicology and Experimental Medicine, Hannover, Germany in accordance with the Declaration of Helsinki and the International Council for Harmonisation Harmonised Tripartite Guideline for Good Clinical Practice. All subjects gave written informed consent.

## Results

### Study population

For the entire clinical trial, 106 subjects were screened. Fifty-seven volunteers were eligible for inclusion and 28 of these were randomly assigned to the placebo group^15^. All subjects were white males with an average age of 32.4 ± 8.4 years, normal BMI (24.9 ± 3.0 kg/m^2^) and normal lung function (FEV_1_: 4.79 ± 0.78 L; FVC: 6.02 ± 0.99 L; FEV_1_/FVC: 0.80 ± 0.04), and had a smoking history of 15.6 ± 17.0 pack-years. Demographic details have been published previously^15^. While females were allowed at a later stage by protocol amendment after conducting required toxicology studies, enrolment was accomplished with male participants only. Of these 28 subjects randomized to the placebo group, three did not complete the treatment and experimental period due to adverse events (AEs) (respiratory tract infection (n = 1), and procedural-related AEs (n = 2)) resulting in 25 completers. Three BAL samples and 17 biopsy samples could not be sequenced or analysed due to the insufficient quality of the samples. Two BAL and two biopsy samples were excluded from further analysis, because they were identified as outliers in the PCA (Fig. 3). A detailed overview of subjects per outcome variable with the primary reason for exclusion or missing value is provided in Table 1.

**Table 1:** Sample overview with primary reason for exclusion.

	BAL (n = 28)	Biopsy (n = 28)
Samples before challenge (baseline)	25	47^1^
Discontinued and missed procedure^2^	1	2^3^
Samples could not be sequenced	1	5
Samples could not be analysed^4^	0	0
Samples excluded from analysis (Fig. 3)	1	0
Samples after saline challenge (saline)	23	20
Discontinued and missed procedure^2^	3	3
Samples could not be sequenced	1	3
Samples could not be analysed^4^	0	1
Samples excluded from analysis (Fig. 3)	1	1
Samples after endotoxin challenge (LPS)	24	16
Discontinued and missed procedure^2^	3	3
Samples could not be sequenced	1	7
Samples could not be analysed^4^	0	1
Samples excluded from analysis (Fig. 3)	0	1

*BAL* bronchoalveolar lavage, *LPS* lipopolysaccharide, *n* number of subjects.

^1^As mentioned in methods, two biopsies per subject were collected at baseline.

^2^Reasons for discontinuation of subjects were respiratory tract infections or procedure-related adverse events.

^3^Because two subjects missed the procedure for biopsy collection at baseline, in total four samples are missing.

^4^Samples excluded from further analysis, because of bad quality (FASTQC).

### Cellular response

**Figure 2:**
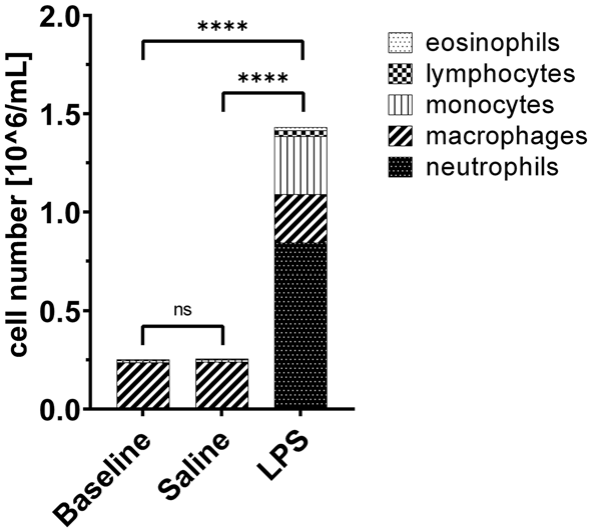
Mean cell counts by cell type in bronchoalveolar lavage at pre-challenge baseline and following LPS or saline challenge for placebo-treated participants. Data are given as placebo group mean. Analysis of variance (ANOVA) followed by Tukey post hoc test was used for statistical comparison. ****p < 0.0001, *LPS* lipopolysaccharide, *ns* not significant.

Main efficacy outcomes, including differential cell counts, cytokines and chemokines in BAL, B1R expression in lung biopsies and inflammatory changes assessed by magnetic resonance imaging, have been described previously^15^. High numbers of inflammatory cells in the airways were induced, dominated by neutrophils after LPS, but not after saline challenge (Fig. 2, Supplementary Fig. 1). Correspondingly, concentrations of CXCL8, albumin and total protein increased in BAL samples after LPS challenge compared to saline control^15^.

### Principal component analysis

**Figure 3:**
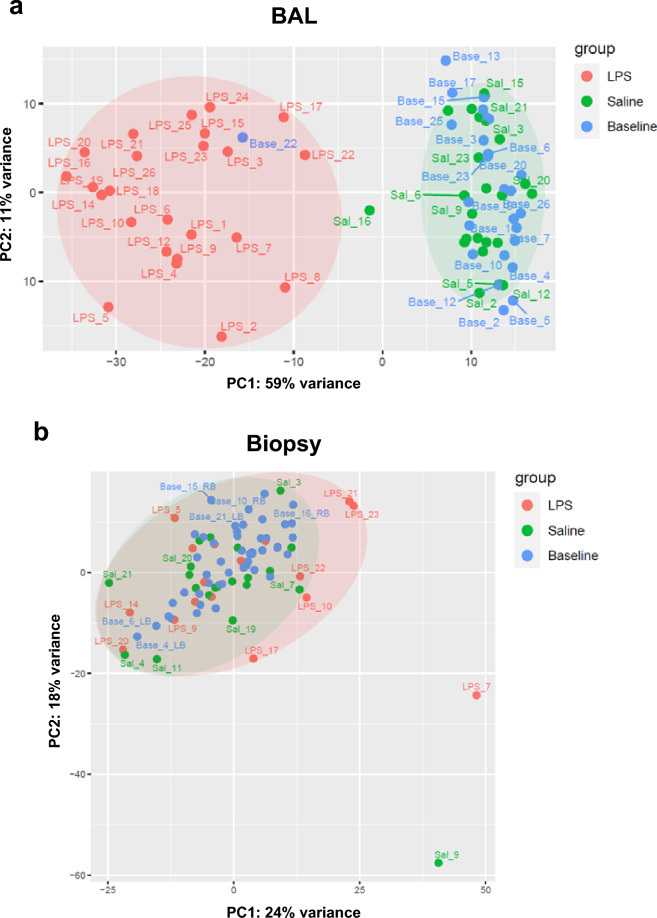
Principal component analysis, depicting clustering of sequenced cells in (**a**) BAL and (**b**) biopsy samples at pre-challenge baseline (blue, BAL: n = 25, biopsy: n = 47) and following LPS (red, BAL: n = 24, biopsy: n = 16) or saline (green, BAL: n = 23, biopsy: n = 20) challenge. *BAL* bronchoalveolar lavage, *Base* baseline, *LPS* lipopolysaccharide, *PC* principal component, *Sal* Saline.

In BAL cells, the first principal component clearly separates the homogenous cluster of baseline and saline samples from the more heterogenous cluster of the LPS-challenged samples (Fig. 3a). Transcriptomic analysis of cells in biopsy samples did not result in group-specific clustering (Fig. 3b). Two BAL samples (Base_22 and Sal_16) and two biopsy samples (Sal_9 and LPS_7) highly differed from their cluster groups. Neither AE profiles nor cell distribution showed abnormalities compared to the other subjects or samples, respectively (data not shown). Therefore, the most likely reason for these outliers was technical variations during the sequencing process. Accordingly, these identified outliers were excluded from further analyses related to differential gene expression and gene set enrichment analyses (Table 1).

### Differential gene expression analysis

**Figure 4:**
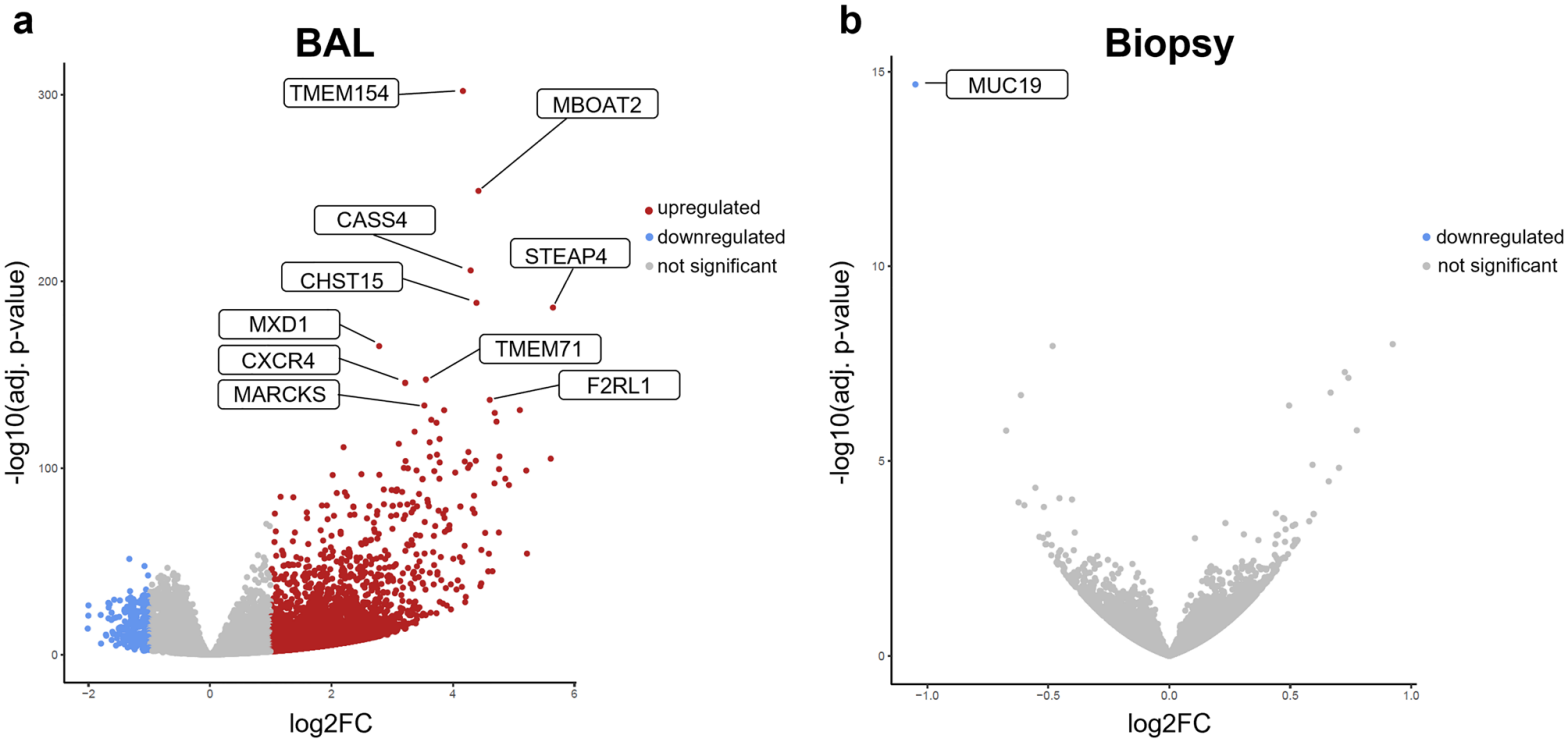
Volcano plot for differential expression analysis results for (**a**) BAL and (**b**) biopsy samples, post LPS (BAL: n = 24, biopsy: n = 16) vs. post saline (BAL: n = 23, biopsy: n = 20) challenge. Differentially expressed genes are highlighted in red for upregulated genes (adjusted p-value ≤ 0.05, log2FC ≥ 1, BaseMean ≥ 2) and in blue for downregulated genes (adjusted p-value ≤ 0.05, log2FC ≤ − 1, BaseMean ≥ 2). The ten most significant genes are labelled with their official gene abbreviations according to ENSEMBL^27^. *BAL* bronchoalveolar lavage, *LPS* lipopolysaccharide, *log2FC* log twofold change.

Differential gene expression analysis resulted in 6557 DEGs (adjusted p-value ≤ 0.05, BaseMean ≥ 2, |log2FC|≥ 1) in BAL (6316 upregulated DEGs and 241 downregulated DEGs), but only one downregulated DEG in biopsy samples after LPS challenge compared to saline challenge (Fig. 4). Among the ten most significant genes in BAL were e.g. STEAP4 (upregulated in inflammatory arthritis and co-localized with macrophages^31^), CXCR4 (involved in AKT signalling cascade^32^, role in regulation of cell migration^33^, mediates LPS-induced inflammatory response^34^) or F2RL1 (synonym: PAR2; modulates human neutrophil cytokine secretion and induces expression of cell adhesion molecules^35,36^, enhances killing of E. coli by human leucocytes^36^, induces dendritic cell maturation^37^). MUC19, the only downregulated DEG in cells of biopsy samples, is a cysteine-rich mucin, which is usually secreted by glandular mucosal cells in airway tissue from healthy individuals^38,39^. Detailed results for each gene of all BAL and biopsy samples are provided in Supplementary Tables 1–4, including calculated |log2FC| with corresponding adjusted p-values and regularized (r)log-normalized counts.

### Gene set enrichment analysis

**Figure 5:**
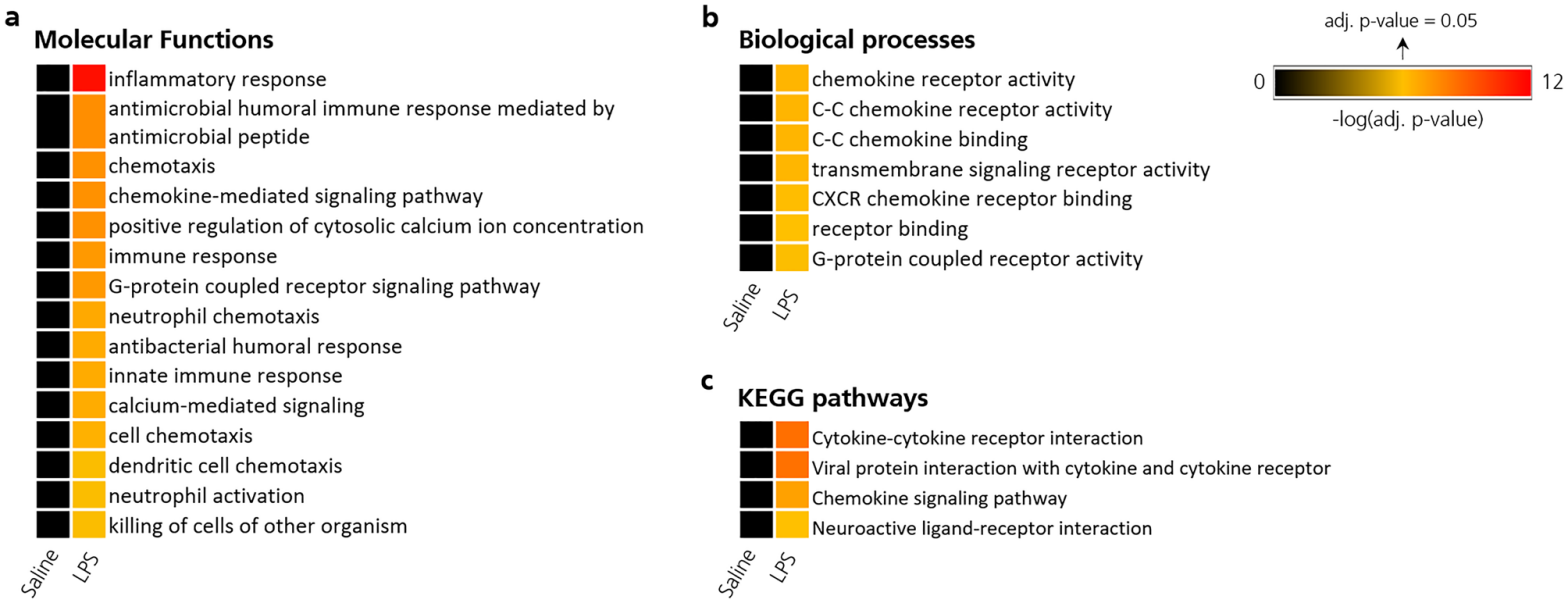
Gene set enrichment analysis using DAVID^28,29^, depicting enriched (**a**) molecular functions, (**b**) biological processes and (**c**) KEGG pathways^40^ in BAL post LPS challenge (n = 24) vs. post saline challenge (n = 23). *BAL* bronchoalveolar lavage, *KEGG* Kyoto Encyclopedia of Genes and Genomes, *LPS* lipopolysaccharide.

**Figure 6:**
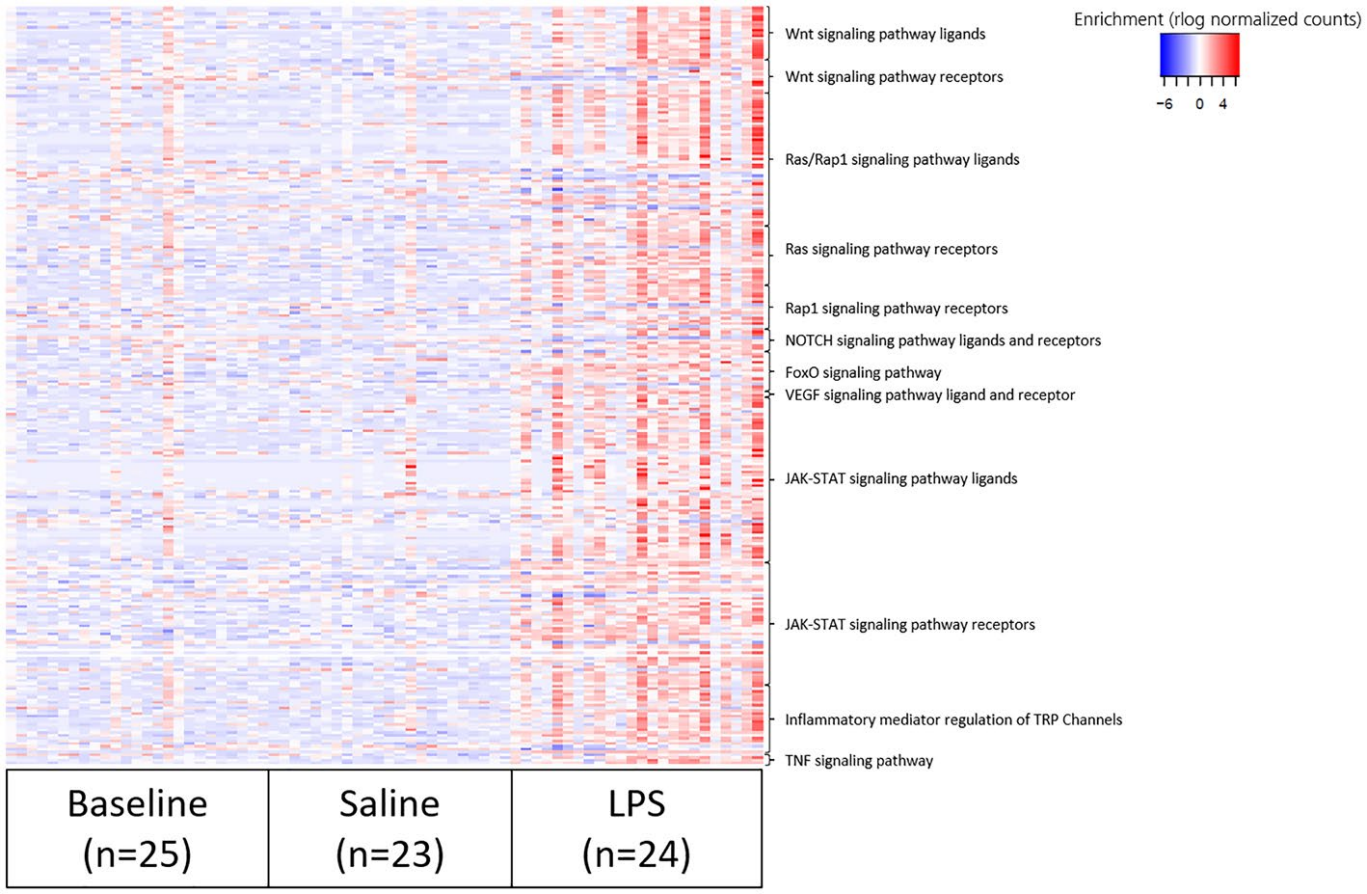
Ligands and receptors of selected pro-inflammatory pathways in the LPS challenge model. The heatmap includes regularized log (rlog) transformed normalized counts, calculated by DeSeq2^26^, of BAL collected at baseline (n = 25), post saline challenge (n = 23) and post LPS challenge (n = 24). *BAL* bronchoalveolar lavage, *LPS* lipopolysaccharide.

Gene set enrichment analysis with upregulated genes in BAL (|log2FC|≥ 3, adjusted p-value ≤ 0.05) using DAVID^28,29^ revealed multiple enriched molecular functions such as “*inflammatory response”*, “*antimicrobial humoral immune response mediated by antimicrobial peptide”* and “*chemotaxis”* (Fig. 5a). Enriched biological processes were, among others, “*chemokine receptor activity”, “C–C chemokine receptor activity”* and “*C–C chemokine binding”* (Fig. 5b). The most significant upregulated pathway in cells of BAL following LPS challenge was “*Cytokine-cytokine receptor interaction”* (Fig. 5c). As expected, ligands and receptors of the pro-inflammatory Wnt-, Ras-, Rap1-, VEGF- and JAK-STAT-signalling pathways were upregulated after segmental LPS challenge compared to saline challenge as well as compared to baseline (Fig. 6). The heatmap depiction revealed high inter-subject variation in intensity of inflammatory pathway upregulation (Fig. 6).

### Comparison of transcriptomic data from the LPS challenge model with respiratory diseases

**Figure 7:**
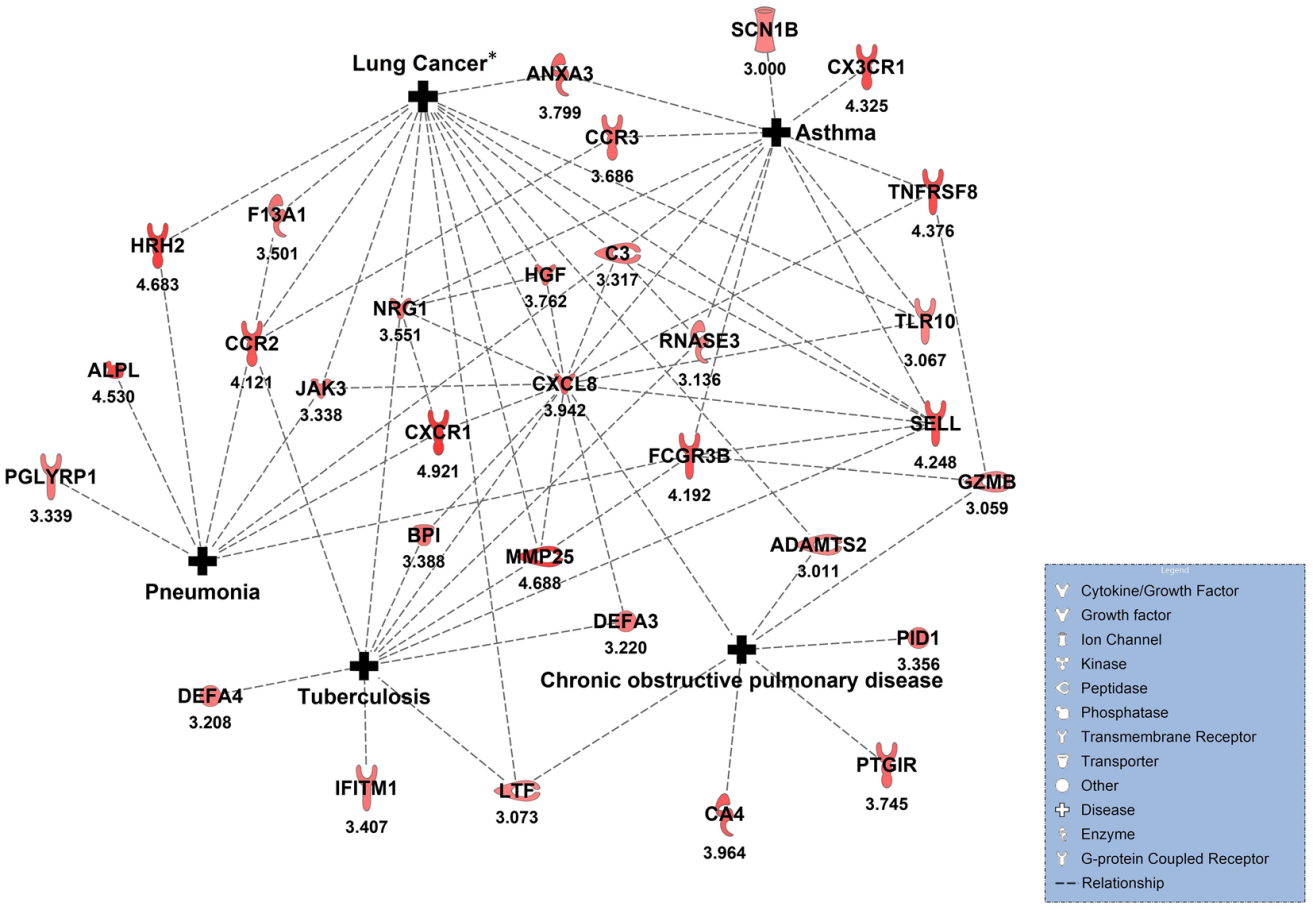
Differentially expressed genes (|log2FC|≥ 3, adjusted p-value ≤ 0.05) assigned to the respective diseases according to Ingenuity Knowledge Base^30^ are shown. Numbers represent the |log2FC| in BAL comparing LPS to saline challenge. *Not all lung cancer genes are shown as it would overlay the figure. The entire gene list is given in Supplementary Table 5. *BAL* bronchoalveolar lavage, *log2FC* log twofold change, *LPS* lipopolysaccharide.

A total of 92 identified DEGs (|log2FC|≥ 3, adjusted p-value ≤ 0.05) were significantly related to the five most prevalent respiratory diseases: COPD, asthma, pneumonia, tuberculosis and lung cancer (Fig. 7). The top hub gene based on connectivity with other network members was CXCL8. Additional, disease-related chemokines such as CCL3L1, CXCL1 or CXCL6 and chemokine receptors such as CXCR1, CX3CR1, CCR2 or CCR3 were identified.

**Figure 8:**
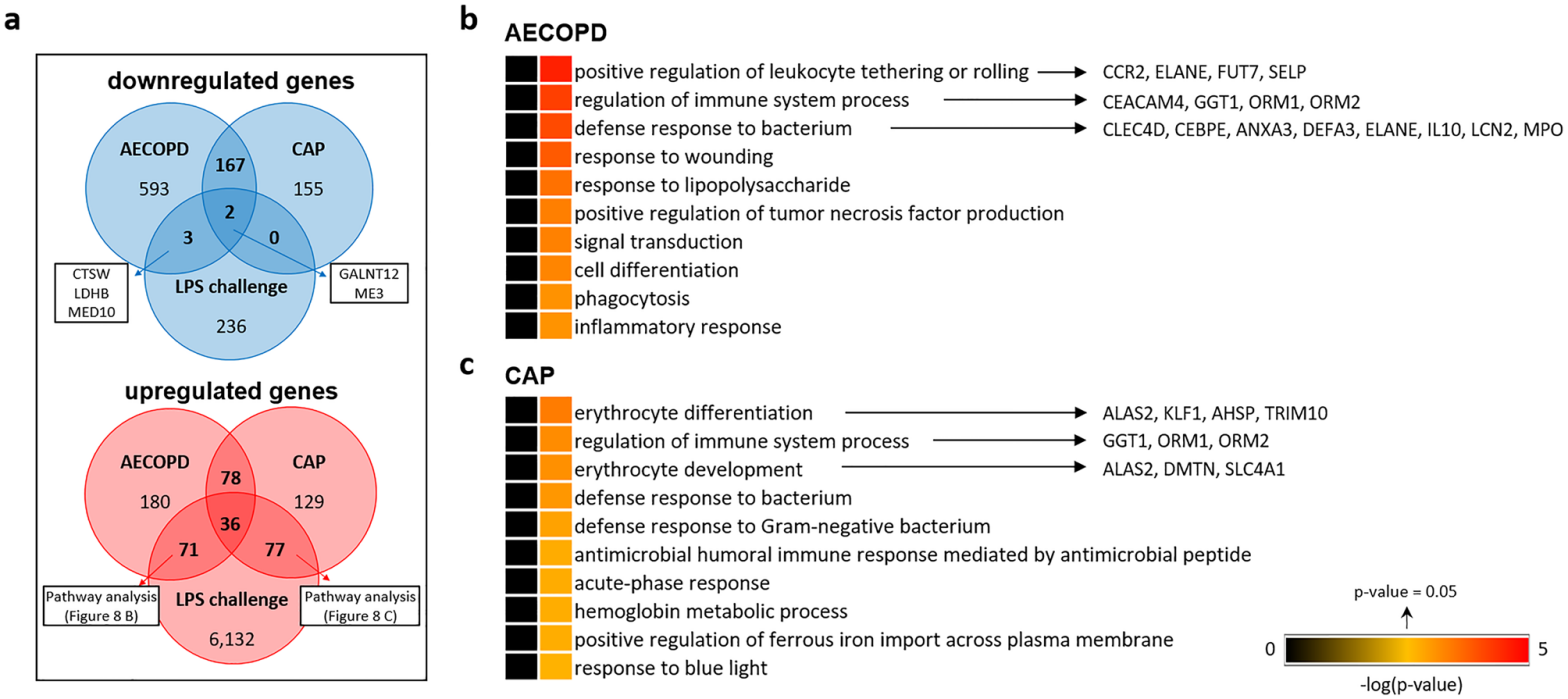
Overlapping properties between BAL obtained post segmental LPS challenge and PBMCs of patients suffering from AECOPD or CAP. (**a**) Venn diagram, depicting numbers of matching upregulated or downregulated DEGs of BAL post LPS challenge and PBMCs of patients with AECOPD or CAP, respectively. Biological processes using upregulated DEGs of the LPS challenge model that are also upregulated in (**b**) AECOPD and (**c**) CAP. *AECOPD* acute exacerbations in chronic obstructive pulmonary disease, *BAL* bronchoalveolar lavage, *CAP* community-acquired pneumonia, *DEG* differentially expressed gene, *LPS* lipopolysaccharide, *PBMC* peripheral blood mononuclear cell.

Furthermore, we compared DEGs following LPS challenge with previously reported transcriptomic analysis. Bertrams et al. identified 1621 genes that were significantly regulated in peripheral blood mononuclear cells (PBMCs) in either of the comparisons AECOPD vs. healthy, CAP vs. healthy or AECOPD vs. CAP^41^. Compared to healthy subjects, 765 protein-coding genes in AECOPD and 324 protein-coding genes in CAP were downregulated (log2FC ≤ − 0.58), whereas 365 genes or 320 genes were upregulated (log2FC ≥ 0.58), respectively (Fig. 8a). Comparing these results to our study following LPS challenge, 29.3% (≙ 107 genes) or 35.3% (≙ 113 genes) of genes were similarly upregulated compared with AECOPD or CAP, respectively. Thirty-six upregulated genes overlapped between AECOPD, CAP and after LPS challenge. In contrast, only five downregulated genes (CTSW, GALNT12, LDHB, ME3, MED10) in AECOPD and two downregulated genes (GALNT12, ME3) in CAP were also downregulated in BAL cells upon LPS challenge (Fig. 8a). Finally, we matched upregulated genes in AECOPD or CAP with LPS for gene set enrichment analysis. Analysis with AECOPD-relevant genes revealed different biological processes such as “*positive regulation of leukocyte tethering or rolling”, “regulation of immune system process”* or “*defence response to bacterium”* with involved genes such as CCR2, IL-10 or MPO (Fig. 8b). Analysis of CAP-relevant genes resulted in significant upregulated biological processes such as “*erythrocyte differentiation”, “regulation of immune system process”* or “*erythrocyte development”* with involved genes such as ALAS2, TRIM10, ORM1 or ORM2 (Fig. 8c).

## Discussion

Segmental endotoxin challenge in humans was first described by O’Grady et al. and demonstrated to safely cause a dose-dependent cell influx, in particular neutrophilia, and increased inflammatory cytokines in BAL^12^. Our study with analysis of transcriptomic data from BAL cells and mucosal biopsies adds to a more in-depth characterization and understanding of the LPS challenge model. Using 23 samples from the LPS segment and 24 from the saline segment, this is, to our knowledge, the largest transcriptomic data set from a segmental LPS challenge model to date. As expected, and in accordance with highly increased cell numbers and protein levels, the LPS-induced inflammatory response resulted in 6557 DEGs, of which 96% were found upregulated. This included several alarmins (interleukin (IL)-25, IL-33), cytokines (IL-2, IL-4, IL-5, IL-8, IL-10, IL-13, IL-17A), chemokines (CXCL1, CXCL8, CXCL11, CCL2, CCL3, CCL8) and chemokine receptors (CCR2, CCR4, CCR5) (Supplementary Table 1), which are involved in pro-inflammatory immune pathways and have been selected as potential drug targets for the treatment of asthma or COPD^42,43^. Furthermore, pro-inflammatory transcription factors that are related to the NF-kappa B signalling pathway (NFKB2 or RELB)^44^ or transcription factors associated with COPD (SNAI1, TWIST1, TWIST2^45^, STAT4^46^, TBX21 (synonym: T-bet)^47^) were found upregulated (Supplementary Table 1). The prominent pro-inflammatory response to LPS was also mirrored by gene set enrichment analysis. These revealed upregulated molecular functions such as “*Inflammatory response”* or “*antimicrobial humoral immune response mediated by antimicrobial peptide”,* enriched biological processes such as “*chemokine receptor activity”*, and upregulated pro-inflammatory pathways such as “*Wnt signaling pathway”, “Ras signaling pathway” or “JAK-STAT signaling pathway”* (Figs. 5 and 6). A heatmap depiction of ligands and receptors involved in several pro-inflammatory pathways elucidated high inter-subject variability (Fig. 6). Variable inflammatory responses are an important feature of LPS challenge in humans^10^, offering the opportunity to identify subgroup-specific gene clusters that predict high or low responders to medication.

In contrast to BAL, no DEGs in response to LPS in cells from biopsy samples except one downregulated gene (MUC19) were observed. One reason for the low number of DEGs could be that bronchial epithelial cells have prominent functions especially during the early phase of the immune response. The main response may have returned to pre-challenge baseline 24 hours post segmental endotoxin instillation. Another explanation for the small number of DEGs in biopsy samples compared with BAL could be that no such neutrophil and inflammatory cell recruitment into the mucosal compartment was observed (Fig. 2) and cellular composition in lung tissue did not change significantly during inflammation.

Transcriptomic analysis of airway samples after LPS challenge has enabled the identification of DEGs that were upregulated in response to LPS challenge and were previously described to play a role in COPD, asthma, pneumonia, tuberculosis, and/or lung cancer (Fig. 7). Targets for treatment of associated respiratory tract diseases based on DEGs that have been identified in our study might therefore be valuable candidates for efficacy studies using the LPS challenge model. Corresponding proteins from many of these DEGs have already been identified as drug targets for the treatment of various diseases such as COVID-19, asthma or rheumatoid arthritis (e.g., CCR2, CCR3, CXCL8, JAK3, HRH, CA4, see clinicaltrials.gov). The hub gene based on connectivity with other network members was CXCL8, a pro-inflammatory cytokine, which is linked to the diseases COPD, asthma, tuberculosis and lung cancer^48–50^. Other disease-related genes were e.g. ANXA3 (regulates NLRP3 inflammasome activity and promotes LPS-induced inflammatory response in bronchial epithelial cells^51^), ADAMTS2 (involved in the emphysema phenotype of COPD^52^, and involved in cleavage of various substrates from the extracellular matrix, growth factors or cytokines^53^) and MMP25 (increased levels in lung tissue and induced sputum of patients with COPD^54^, matrix metalloproteinases accelerate pro-inflammatory processes in respiratory diseases^55^). Furthermore, chemokines (CCL3L1, CXCL1 or CXCL6) and chemokine receptors (CXCR1, CX3CR1, CCR2 or CCR3) were identified, whose interaction contributes to recruitment of pro-inflammatory cells and related inflammation in respective diseases. This indicates that some pro-inflammatory pathways and mechanisms that are found to be relevant in respiratory diseases are reflected by BAL cell transcriptome post segmental LPS challenge.

The lower respiratory tract of patients with COPD is often colonized with gram-negative bacteria^56^ as a source of LPS. This microbiome could contribute to progression and exacerbations of COPD^57–59^. CAP is not only caused by the gram-positive bacterium Streptococcus pneumoniae^3^, but also by viruses and gram-negative bacteria^4^. However, it is not clear which aspects of AECOPD and CAP are potentially reflected by segmental LPS challenge. Bertrams et al. recently published a list of potential biomarker genes in pneumonia and AECOPD^41^. We analysed whether DEGs in PBMCs of patients with CAP or AECOPD compared to healthy controls are also differentially expressed in BAL cells post LPS challenge in healthy smokers. Interestingly, only five matching downregulated genes in AECOPD (CTSW, GALNT12, LDHB, ME3, MED10) and two in CAP (GALNT12, ME3) were found. One of the five downregulated genes in AECOPD was CTSW, which promotes viral entry^60^ and may have a specific function during target cell killing by CD8^+^ T cells and NK cells^61^. Matching genes between AECOPD and CAP were GALNT12 (deficiency in mice leads to decreased cell proliferation, migration and invasion^62^) and ME3 (important for insulin secretion in pancreatic β-cells^63^, knockdown of ME2 suppresses lung tumour growth^64^ and is a potential therapeutic drug target for cancer^65^). In contrast, 107 genes (29.3%) in AECOPD and 113 genes (35.3%) in CAP were matching with upregulated genes in BAL cells after LPS challenge (Fig. 8a). Thirty-six of these identified genes overlapped between AECOPD and CAP, suggesting similar regulation patterns in these two diseases^41^. Expression patterns in whole blood revealed major differences compared to lung tissue from patients with COPD^66^. Still, we were able to find overlapping genes between cells derived from challenged BAL and PBMCs, which could potentially help to identify matrix-independent biomarkers. Matching genes between AECOPD or CAP and the LPS challenge model were assigned to several biological processes such as “*positive regulation of leukocyte tethering or rolling”, “regulation of immune system process”* or “*defence response to bacterium*” for AECOPD, and “*erythrocyte differentiation”, “regulation of immune system process*” or “*erythrocyte development"* for CAP (Fig. 8). Because clinical development of drugs for prevention or treatment of COPD exacerbations is demanding, complex and time-consuming, the LPS model might offer the option to generate proof-of-principle information in early-phase clinical trials given certain similarities of AECOPD and CAP with the LPS challenge model. Our gene set enrichment analysis identified genes or pathways of interest based on gene annotations with known functional information sources (Figs. 7 and 8). In addition, other LPS-regulated proteins, such as CXCR2, IL1R1/IL1R2, MAPK11, MCP-1 or PDE4 (Supplementary Table 1), could also be potential targets. Interestingly, aforementioned molecules have already been targets in previous inhaled or segmental LPS challenge studies, all with a positive study outcome^67–72^. In addition to associations of DEGs to respiratory diseases or infection-driven exacerbations of respiratory diseases, our data might also allow for a more detailed analysis into epigenetic and cytoskeletal remodelling or LPS-induced immune tolerance^73^.

This study carries limitations. Transcriptomic data were derived from healthy smokers at baseline and following LPS challenge. Smokers have changes in airway inflammatory cells compared with healthy non-smokers. For example, increased numbers of inflammatory cells in the bronchial mucosa and structural changes such as increased thickness of the tenascin and laminin layers have been described^74^. Also, a significant percentage of smokers with preserved pulmonary function have been identified to suffer pre-COPD^75^. Therefore, DEGs after LPS challenge might be different in current smokers compared to healthy non-smokers. However, the inflammatory cytokine response of smokers to LPS challenge is similar (e.g. IL-8, TNF-α) or only slightly increased (e.g. IL-1β) compared with healthy subjects^76^. Also, current smoking in COPD did not affect airway mucosal inflammation in COPD compared with ex-smokers^77^. Since cigarette smoke, which contains LPS^78,79^, is the most common cause of COPD^5^, and 38% of patients with COPD remain current smokers^80^, smoking subjects are closer to the phenotype of COPD patients compared with healthy non-smoking subjects. While it might be warranted to enrol smoking volunteers for studies testing potential drugs against COPD or AECOPD, this must be balanced against potential increases in variability of inflammatory and structural cells. Furthermore, gender-specific differences in the immune response to airway challenges could occur, as documented in several murine models^81,82^. Since only male subjects were recruited in this study due to reasons given above, no gender-specific conclusions can be drawn.

In summary, our study provides comprehensive data on DEGs from BAL cells and mucosal biopsies following LPS challenge in healthy smokers. It furthers our understanding of the LPS challenge model about similarities with respiratory diseases in general and infection-triggered respiratory insults such as exacerbations in particular.

### Supplementary Information

Supplementary Figure 1.

Supplementary Tables.

## Data Availability

To ensure independent interpretation of clinical study results and enable authors to fulfil their role and obligations under the ICMJE criteria, Boehringer Ingelheim grants all external authors access to relevant clinical study data. In adherence with the Boehringer Ingelheim Policy on Transparency and Publication of Clinical Study Data, scientific and medical researchers can request access to clinical study data after publication of the primary manuscript in a peer-reviewed journal, regulatory activities are complete and other criteria are met. Researchers should use the https://vivli.org/ link to request access to study data and visit https://www.mystudywindow.com/msw/datasharing for further information. Count data files obtained by RNASeq data analysis is published in the ArrayExpress Archive of Functional Genomics Data (http://www.ebi.ac.uk/arrayyexpress): E-MTAB-13318.

## Abbreviations

AECOPD: Acute exacerbations of COPD
AE: Adverse event
B1R: Bradykinin 1 receptor
BAL: Bronchoalveolar lavage
BMI: Body mass index
CAP: Community-acquired pneumonia
COPD: Chronic obstructive pulmonary disease
DEG: Differentially expressed gene
FEV_1_: Forced expiratory volume in 1 s
FVC: Forced vital capacity
IL: Interleukin
Log2FC: Log twofold change
LPS: Lipopolysaccharide
N: Number of subjects
PBMC: Peripheral blood mononuclear cell
PCA: Principal component analysis
RNA-seq: RNA-sequencing
