# Supplementary material for: Transcriptomic characterization of the human segmental endotoxin challenge model
Source: Sci Rep. 2024 Jan 19;14:1721. doi: 10.1038/s41598-024-51547-0 (PMC10798985; doi:10.1038/s41598-024-51547-0)
Supplement: Supplementary file 1 — Supplementary Figure 1. [file 41598_2024_51547_MOESM1_ESM.pptx]

## Slide 1
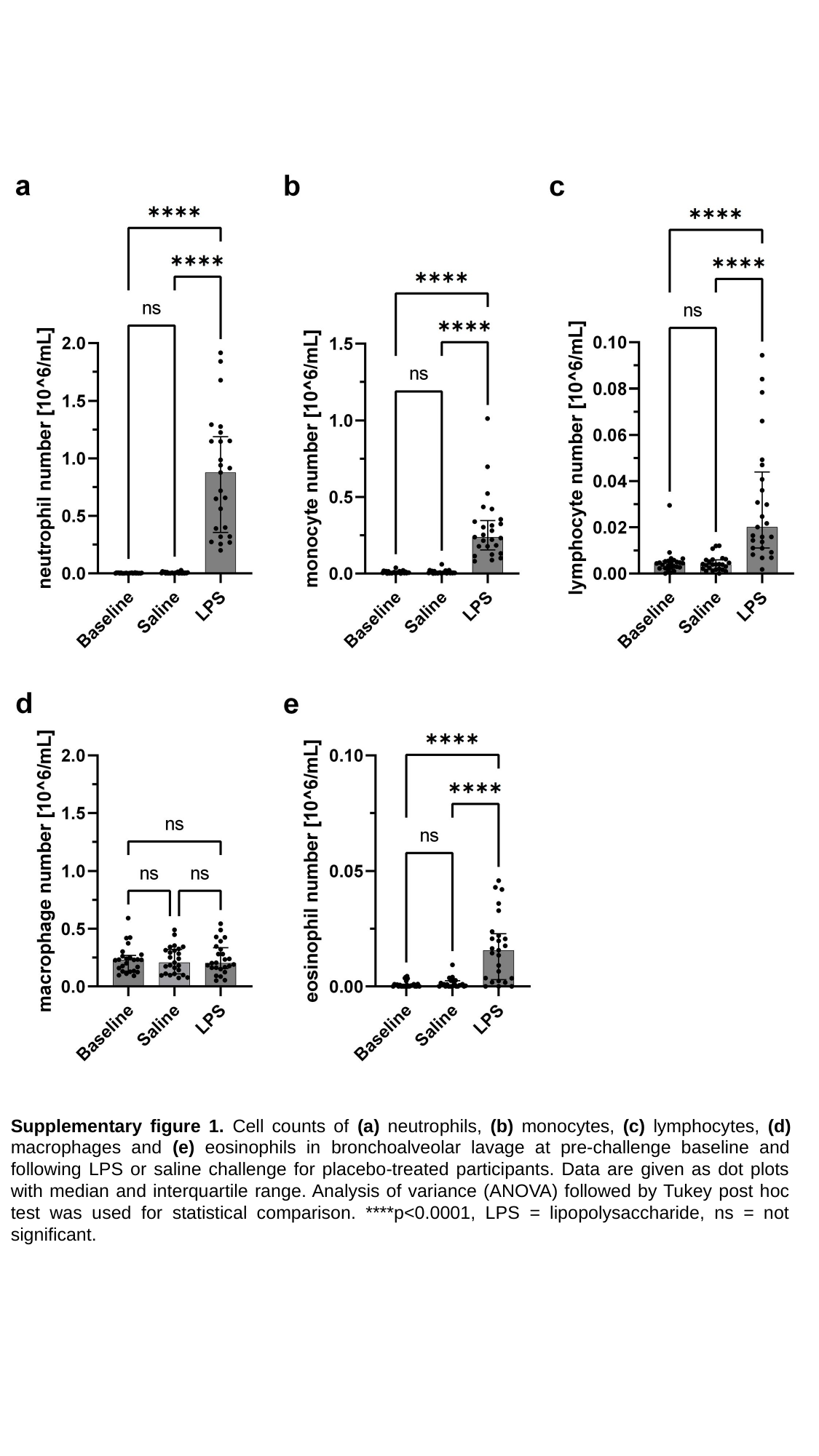

Supplementary figure 1. Cell counts of (a) neutrophils, (b) monocytes, (c) lymphocytes, (d) macrophages and (e) eosinophils in bronchoalveolar lavage at pre-challenge baseline and following LPS or saline challenge for placebo-treated participants. Data are given as dot plots with median and interquartile range. Analysis of variance (ANOVA) followed by Tukey post hoc test was used for statistical comparison. ****p<0.0001, LPS = lipopolysaccharide, ns = not significant.
